# The Effects of Scan Body Geometry on the Precision and the Trueness of Implant Impressions Using Intraoral Scanners: A Systematic Review

**DOI:** 10.3390/dj13060252

**Published:** 2025-06-05

**Authors:** Roksana Mohajerani, Shirin Djalalinia, Marzieh Alikhasi

**Affiliations:** 1 School of Dentistry, Tehran University of Medical Sciences, Tehran 1416634793, Iran; r-mohajerani@student.tums.ac.ir; 2 Development of Research and Technology Center, Deputy of Research and Technology, Ministry of Health and Medical Education, Tehran 1467664961, Iran; 3 Dental Research Center, Dentistry Research Institute, Tehran University of Medical Sciences, Tehran 1416634793, Iran

**Keywords:** dental implants, intraoral scanner, scan body geometry, digital impressions, accuracy, trueness, systematic review

## Abstract

**Background/Objectives:** Accurate implant impressions are critical for capturing the three-dimensional (3D) spatial positioning of implants. Digital workflows using intraoral scanners (IOSs) and scan bodies offer distinct advantages over conventional elastomeric techniques. However, the geometry of scan bodies may influence the precision and trueness of IOS-acquired data, and optimal design parameters remain undefined. This systematic review aims to evaluate the effects of scan body geometry on the trueness of digital implant impressions captured using IOSs. **Methods**: A systematic search was conducted across PubMed, Scopus, EMBASE, Web of Science, the Cochrane Library, and Google Scholar up to 25 February 2025. Eligible studies assessed the impact of scan body geometry on the accuracy of implant-level impressions acquired with IOSs. Study quality was assessed using the Quality Assessment Tool for In Vitro Studies of Dental Materials (QUIN). **Results**: Twenty-eight studies were included, of which twenty-six were in vitro. The included studies, published between 2020 and 2025, demonstrated that variations in macro- and micro-geometries influenced both linear and angular trueness. Cylindrical designs with optimal dimensions generally outperformed cuboidal or spherical forms. Structural modifications, such as rigid bar extensions and surface facets, often improved scan accuracy. Some hybrid or modified designs performed comparably to conventional scan bodies. According to QUIN, 27 studies were moderate quality and one had high quality. **Conclusions**: Scan body geometry affected the accuracy of intraoral implant digital impressions. Designs featuring rigid extensions or simplified geometries improve trueness and precision. Further standardized clinical studies are needed to define optimal design features and validate current in vitro findings.

## 1. Introduction

Accurate replication of implant position is essential for the long-term success of implant-supported prostheses [1]. Conventional methods rely on impression copings and elastomeric materials [2]; but, the advent of intraoral scanners (IOSs) has introduced more efficient digital workflows. These digital techniques offer reduced chair time, improved patient comfort, and eliminate the need for physical storage [3]. Clinical studies have shown that digital implant impressions are comparable in accuracy to conventional techniques [4,5,6].

Central to digital implant workflows is the scan body, which acts as the reference point for digitizing implant positions [7]. Traditional impressions are susceptible to distortion, material shrinkage, and technique sensitivity [8], whereas IOS workflows, using scan bodies, allow for high-resolution, reproducible impressions with minimal discomfort [8,9,10]. This shift has contributed to the widespread clinical adoption of IOSs [10].

Scan bodies differ in shape, size, surface features, and material composition—all of which can influence scan quality [9]. Many designs include anti-rotational features such as flat facets or beveled surfaces to replicate the implant’s internal connection. However, the optimal configuration for these features remains unclear [11,12]. Additionally, the choice between metal and polymer materials and the effects of surface texture on scan accuracy remain subjects of ongoing research [9].

The performance of IOSs is generally evaluated by precision (repeatability of scans) and trueness (deviation from the true value) [13,14]. While several systematic reviews have explored the overall accuracy of IOSs [1,4,14,15], few have directly examined how scan body design influences outcomes. Existing meta-analyses have yet to provide consensus regarding the ideal geometry, dimensions, and surface characteristics of scan bodies [16,17]. Given the increasing use of digital impressions and the growing diversity of scan bodies, a targeted systematic review is needed. This review aimed to evaluate the impact of scan body geometry—including macro-shape, micro-features, and structural attachments—on the accuracy of digital implant impressions acquired with IOSs. By synthesizing the current evidence, we seek to guide clinicians toward more informed scan body selection for improved clinical outcomes.

## 2. Materials and Methods

### 2.1. Review Design and Protocol

This systematic review was conducted according to the 2020 Preferred Reporting Items for Systematic Reviews and Meta-Analyses (PRISMA) guidelines [18]. This systematic review was not registered in a public repository. The research question was developed using the PICO framework: P (Population) included implant-supported restorations; I (Intervention) referred to the use of IOSs in conjunction with scan bodies; C (Comparison) involved different scan body geometries; and O (Outcome) was the accuracy and trueness of digital implant impressions. To ensure a comprehensive, broad, and sensitive literature search capable of capturing all potentially relevant studies—including those reporting rarer or alternative outcome measures—we did not incorporate specific outcome terms (e.g., accuracy, precision, trueness) into the search strategy. This approach, informed by similar protocols and expert input, avoided undue restrictions on study inclusion.

### 2.2. Eligibility Criteria

#### 2.2.1. Study Types

Eligible studies included peer-reviewed in vitro and in vivo research, randomized and non-randomized controlled trials, cohort studies, and case-control studies. Exclusion criteria included duplicate reports, conference abstracts without full data, and studies that failed to report relevant scan body geometry data. No restrictions were placed on publication year, language, or the age of study subjects.

#### 2.2.2. Participants

Studies involving dental implants scanned intraorally using scan bodies were included. This encompassed patient-based, model-based, or typodont studies evaluating the impact of scan body design on impression accuracy.

#### 2.2.3. Interventions and Comparisons

The primary intervention was the use of scan bodies during IOS-acquired impressions. Comparisons were made across different scan body geometries, including variations in shape (e.g., cylindrical, conical, hexagonal), dimensions, surface modifications, and adjunctive features.

#### 2.2.4. Outcomes

The primary outcome was trueness—defined as the closeness between the measured and actual implant position. Studies employing any validated measurement methodology were eligible.

### 2.3. Information Sources and Search Strategy

**Figure 1:**
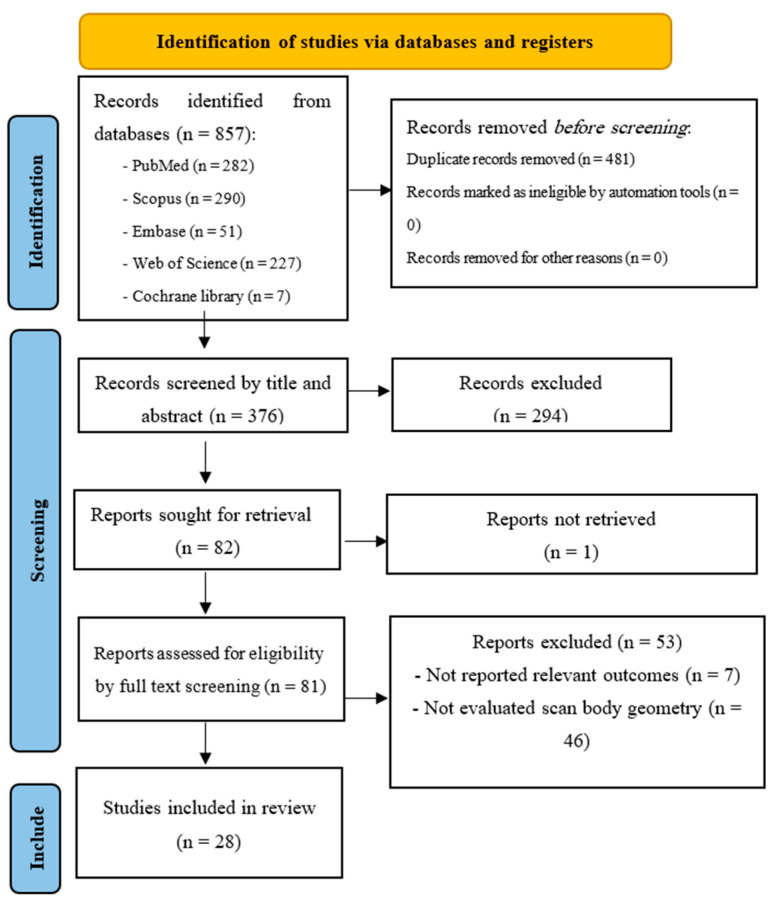
Study selection process flow diagram. No further study was found through Google Scholar.

A comprehensive literature search was performed across PubMed/MEDLINE, Scopus, EMBASE, Web of Science, and the Cochrane Library. The search was completed on 25 February 2025. Keywords and Medical Subject Headings (MeSH) related to IOSs, scan body geometry, and implant accuracy were used (see Table S1). Manual searches of references from eligible studies and systematic reviews were also performed. The first 300 Google Scholar results were screened to identify gray literature [19]. A PRISMA flow diagram (Figure 1), was used to document the selection process.

### 2.4. Study Selection and Data Extraction

All records were imported into Rayyan systematic review software (a continuously updated, web-based platform with no fixed version number) for blinded, duplicate screening [20]. Two independent reviewers performed title, abstract, and full-text screening. Discrepancies were resolved via discussion or by consulting a third reviewer. Data extraction was completed using a pre-piloted Excel form. Extracted data included study title, year, scan body material, scanner system, implant position and angulation, scan body geometry (e.g., height, diameter), torque values, sterilization procedures, measurement methods, and main findings. Inter-reviewer agreement for data extraction was evaluated using Cohen’s Kappa (κ = 0.86), indicating substantial agreement.

### 2.5. Risk of Bias Assessment

Risk of bias was assessed using the QUIN tool [21], which evaluates 12 methodological domains including sample size justification, operator information, randomization, blinding, and outcome reporting. Each criterion was scored from 0 (inadequate) to 2 (adequate). Final scores were converted to weighted percentages, and studies were classified as high (>70%), moderate (50–70%), or low (<50%) quality. Disagreements were resolved by consensus or third-party adjudication.

### 2.6. Data Synthesis

The extracted data were synthesized qualitatively. Due to the heterogeneity in study designs, scan body geometries, and measurement protocols, a meta-analysis was not performed. Instead, a narrative synthesis approach was adopted to compare findings across studies. Descriptive statistics, including mean, standard deviation, and significance values (when available), were extracted for linear and angular trueness. Studies were grouped by geometry type (e.g., cylindrical, cuboidal, modified) and structural characteristics (e.g., extensional features, surface modifications). Where studies used comparable geometry categories and outcomes, patterns were analyzed and discussed.

### 2.7. Ethical Considerations

As a systematic review of previously published literature, no ethical approval was required. All included studies were checked for prior ethical clearance.

## 3. Results

### 3.1. Study Selection

An initial 857 articles were retrieved through the systematic search, with 376 records remaining after duplicates were removed. Title screening led to the exclusion of 244 studies, leaving 132 articles for abstract evaluation. After the abstract screening, 50 additional studies were excluded, and 82 articles were selected for full-text assessment. The full text of one study was unavailable, 46 studies did not report scan body geometries, and 7 studies were excluded for not reporting the outcomes of interest. No further study was found through Google Scholar. Ultimately, 28 studies were included in this systematic review [8,22,23,24,25,26,27,28,29,30,31,32,33,34,35,36,37,38,39,40,41,42,43,44,45,46,47,48] (Figure 1).

### 3.2. Study Characteristics

The studies included in the review were published between 2020 and 2025. Of 28 studies, 26 were in vitro studies, and 2 were in vivo [27,46]. The total sample size across the studies was 339, with subject numbers ranging from 4 to 68. The number of implants used in the studies ranged from 1 to 10. The most common measurement technique employed was the best-fit algorithm (Table 1 and Table S2).

Across the 28 included studies, a wide range of macro-geometries (cylindrical, cuboidal, spherical, hybrid, and tooth-modified bodies) and micro-geometric modifications (rigid bar extensions, extensional structures, additive/subtractive alterations, surface facets, and depression-type features) were evaluated. Cylindrical scan bodies—particularly those with a 5.5 mm diameter and 12 mm height—consistently demonstrated the best linear and angular trueness. In contrast, cuboidal and spherical designs generally showed inferior trueness, with spherical shapes precluding angular measurements. Among micro-modifications, rigid bar extensions and extensional structures yielded the greatest reductions in mean deviation and angular error, whereas additive surface modifications tended to degrade trueness. Other designs, such as surface facets and depression-type bodies, produced mixed but sometimes significant improvements in surface accuracy. A comprehensive summary of these findings is provided in Table 2.

### 3.3. Scan Body Geometry

The studies showed that variations in scan body geometry—whether in macro-shape, micro-features, or adjunctive attachments—can influence both linear and angular accuracy in digital implant impressions. In the only direct comparison of cuboidal versus dome-shaped bodies, Pan et al. reported that the cuboidal design yielded larger mean deviations in model surface trueness (13.9 ± 0.7 µm vs. 10.7 ± 0.2 µm) but similar centroid linear errors and angular center-axis displacements (*p* = 0.495 and *p* = 0.091, respectively). This suggests that while surface fidelity may change, critical angular alignment is preserved [33].

Another study investigated nine cylindrical (4.8–6.5 mm at heights 4, 8, and 12 mm), five cuboidal (cross-sections 3 × 6–5 × 6 mm, heights 8–12 mm), and a spherical scan body in vitro. It found that the 5.5 × 4 mm cylinder achieved the best linear trueness (4.0 ± 2.4 µm), while the 4 × 6 × 6 mm cuboid exhibited the worst (28.8 ± 8.6 µm). Angular trueness was optimal for the 5.5 × 12 mm cylinder (0.013 ± 0.010°) and poorest for the 4 × 6 × 6 mm cuboid (0.178 ± 0.010°). Furthermore, cylinder height (*p* = 0.034), diameter (*p* = 0.001), and their interaction (*p* = 0.007) significantly influenced linear trueness, while angular trueness in cylindrical groups was affected by height (*p* < 0.001), diameter (*p* < 0.001), and interaction (*p* = 0.004) [48] (Table 2 and Table S2).

Comparisons of extensional features consistently favored designs with added structure. In an in vivo study, Huang et al. found that a rigid-bar extension on a flat-sided scan body reduced mean linear deviation from 119.5 ± 83.3 µm to 68.9 ± 31.3 µm (*p* = 0.008) and angular error from 0.75 ± 0.79° to 0.36 ± 0.29° (*p* = 0.049) [27]. Its in vitro counterpart showed that CAD/CAM bodies with extensions achieved a median trueness of 28.5 µm versus 35.9 µm for originals and 38.5 µm for non-extended CAD/CAM models (*p* = 0.001), although pairwise contrasts were non-significant [22].

Lawand et al. demonstrated that subtractive modifications improved angular trueness (0.993 ± 0.062°) compared with non-modified and additively modified bodies (*p* < 0.001), whereas additive changes degraded surface accuracy [41]. For CAD/CAM prototypes, Zhang et al. detected significant intergroup differences (*p* < 0.001) but no consistent superiority among straight, arcuate, or no-extension designs [45] (Table 1).

More nuanced geometry studies underscored the interplay of shape and complexity. In a study by Revilla-León et al., three manufacturer bodies (beveled, polygonal, flat-one-facet) exhibited similar linear errors (4–8 µm) but differed in XZ angular distortion, with NT-Trading outperforming the others, and Dynamic Abutment showing the greatest YZ angle variability [26]. Another study by the same group found no significant linear discrepancies between the two bodies but noted higher XZ angular error in the Elos system versus NT-Trading (*p* < 0.001), highlighting that even single-degree differences can be clinically relevant [28].

Another in vitro study compared four commercial geometries and identified Ticare MG (0.050 ± 0.039 mm, 0.185 ± 0.189°) and Talladium (0.041 ± 0.024 mm, 0.221 ± 0.186°) as most accurate, significantly outperforming ELOS and MG (*p* < 0.01) [31] (Table 2 and Table S2).

Large-scale prototype testing by Meneghetti and colleagues of seven PEEK- and resin-based shapes revealed median 3D deviations ranging from 72.3 µm (SB2) to 190.3 µm (SB5), with angular errors from 0.25° to 0.89° (*p* < 0.001), showing that even subtle surface facets and heights (7–16 mm) markedly alter trueness [34]. Manufacturer comparisons in another study similarly found ELOS (0.041 mm) and TeamZiereis (0.035 mm) significantly more accurate than NT-Trading (0.112 mm) on the X and Z axes (*p* < 0.01) [25] (Table 2 and Table S2).

Hybrid comparisons involving healing abutments showed mixed results. In the study by Yilmaz and colleagues, conventional scan bodies and a healing-abutment-scanpeg system yielded comparable point-to-point linear (0.014–0.043 mm vs. 0.076–0.178 mm) and angular (0.186–0.273° vs. 0.195–0.273°) deviations, supporting both methods for single-implant anterior scans [30]. Furthermore, Ramadan et al. directly contrasted a one-piece Elos Medtech body (0.054 ± 0.001 mm, 0.379 ± 0.023° vert) with a two-piece Neoss HA-SP (0.182 ± 0.004 mm, 1.676 ± 0.073° vert), finding significantly lower linear and angular errors in the one-piece design (*p* < 0.001) [35] (Table 1, Table 2 and Table S2).

Studies of digital versus conventional or healing-jig techniques further showed the effects of geometry. In this regard, Mizumoto et al. showed that both body type and scan method independently influenced distance and angular trueness (*p* < 0.05), with Zimmer Biomet bodies producing less deviation than Dentsply Sirona [23]. In addition, Jung et al. reported that simple scan abutments had higher intra-arch linear deviation than scanning jigs (*p* < 0.05), although inter-arch errors remained under 100 µm [32]. Moslemion et al. determined that Doowon and NT-Trading bodies outperformed DESS in linear (0.05–0.06 mm vs. 0.17 mm) and angular (0.35–0.52° vs. 0.47°) metrics (*p* < 0.001) [24]. In the digital-versus-conventional realm, another study found that short bodies yielded superior platform trueness (37–52 µm vs. 90–128 µm) and angle accuracy (0.11–0.25° vs. 0.31–0.57°; *p* < 0.001) [38] (Table 1, Table 2 and Table S2).

Studies probing specialized features found diverse effects of geometry. Accordingly, Schmidt et al. detected no trueness differences among the three bodies (0.106–0.134 mm), suggesting that some bespoke designs may converge on similar accuracy [29]. In addition, Tan et al. demonstrated significant global distortion variation across four branded bodies (11–42 µm; *p* < 0.001), independent of applied torque [8]. Li and colleagues showed that modified Digital Wings bodies achieved a maximum RMS error of 37.5 µm versus higher errors for Straumann scan bodies (*p* < 0.001) [42]. Also, in another study adding a vertical stop to conventional bodies improved linear errors at 11° conical implants (Δd from 0.182 mm to 0.129 mm; *p* < 0.05) and reduced most angular displacements (*p* < 0.05), though trends varied by implant site [44] (Table 1, Table 2 and Table S2).

Accessory and attachment studies showed various results. Ashry et al. demonstrated that accessories reduced overall 3D deviations from 0.210 ± 0.058 mm to 0.180 ± 0.039 mm (*p* = 0.043) without altering angular errors [40]. Also, Farah et al. evaluated geometric attachments in iTero and OmniCam scans, halving RMS errors (e.g., OmniCam) from 70.8 ± 10.3 µm to 35.2 ± 3.6 µm (*p* < 0.001) [47]. Mesh congruence research found that simpler cylindrical designs (STR, MIS) achieved superior mesh-library alignment (0.019 ± 0.007 mm) compared with more complex shapes (0.029–0.046 mm; *p* < 0.05) [43] (Table 1, Table 2 and Table S2).

Regarding geometric modifications, Uzel et al. observed that proximal slots up to 6 mm substantially increased linear (up to 137 ± 41.7 µm) and angular (up to 2.56 ± 1.88°) deviations, underscoring the risk of excessive feature removal (*p* < 0.05) [37]. Also, Shely et al. compared asymmetrical trapezoid versus cylindrical bodies under lab versus IOS scanning, finding significant linear (0.020–0.135 mm vs. 0.021–0.057 mm) and angular (0.294–1.776° vs. 0.139–2.042°) differences in site-specific errors (*p* < 0.0005) [36]. Another study by Eldabe et al. showed that tooth-modified bodies halved 3D deviations (61.5 ± 42.1 µm vs. 98.0 ± 56.7 µm) and reduced angular error (0.85 ± 0.69° vs. 1.30 ± 1.06°; *p* < 0.033) [46]. Round-depression designs also improved mean surface error from 0.282 ± 0.038 mm to 0.229 ± 0.047 mm (*p* = 0.004) in a complete-arch model [39] (Table 1, Table 2 and Table S2).

### 3.4. Quality Assessment

The methodological quality of the 28 included studies varied, with QUIN scores ranging from 54.5% to 72.7%. Only one study—Moslemion et al.—achieved a low risk of bias, scoring above the 70% threshold (72.7%) [24]. The remaining 27 studies were categorized as having moderate risk (scores between 54.5% and 68.2%) [8,22,23,25,26,27,28,29,30,31,32,33,34,35,36,37,38,39,40,41,42,43,44,45,46,47,48]. Across these moderate-risk studies, several key domains were consistently underreported: detailed sample size calculations; operator credentials and experience; randomization procedures; blinding of outcome assessors; and explicit information on outcomes. In contrast, domains such as clearly stated objectives, detailed methodologies, appropriate group comparison, robust accuracy measurement, statistical analyses, and clear results presentation were well reported (Table S3).

## 4. Discussion

This systematic review demonstrates that scan body geometry plays a pivotal role in the accuracy of digital implant impressions. Geometric characteristics—ranging from macro shape and dimensional parameters to surface features and accessory structures—significantly influenced both linear and angular trueness across diverse scanning conditions.

Macro-shape comparisons showed that cuboidal bodies exhibited larger surface deviations than dome-shaped ones while maintaining similar angular alignment, and that polygonal and beveled designs transferred linear positions accurately but differed in XZ angular error. Manufacturer-specific geometries varied markedly in 3D and 2D displacement, with ELOS A/S and TeamZiereis outperforming NT-Trading. Cylindrical bodies of optimal height and diameter achieved the best linear and angular trueness across diverse morphologies. Hybrid healing–abutment systems performed comparably to conventional scan bodies for single-implant scans, although one-piece designs were superior to two-piece assemblies. Surface modifications and accessories—such as subtractive alterations, accessory parts, and geometric attachments—either improved accuracy or, when overly extensive, degraded trueness. Modest depression-type modifications improved trueness in complete-arch scans.

We found that scan bodies generally improved both linear and angular trueness of digital implant impressions. In our systematic review, multiple studies demonstrated that the addition of extensional features, such as wings or lateral projections, contributed to improved accuracy, likely by enhancing scan body detectability and reference point clarity. For instance, in Farah et al. 2025, when assessing the impact of geometric attachments, we selectively extracted data from the parallel group in order to isolate the pure geometric effect, avoiding confounding from implant angulation [47]. This pattern of enhanced accuracy aligns with the broader literature and reinforces the potential value of structural augmentation in scan body design [49]. Moreover, Gehrke et al. conducted a systematic review of intraoral scanning factors—highlighting scan body placement, structural design, material, color, manufacturing system, IOS type, scanning technique, inter-implant distance, scan span, and implant number—as determinants of digital implant impression precision, particularly in single- and short-span cases [7]. In addition, another systematic review identified multiple influencing factors such as implant angulation, inter-implant distance, scan body characteristics, and operator experience as determinants of impression precision [50]. Furthermore, Sanda et al. highlighted the number of implants, their spatial distribution, and the characteristics of the scan body as factors affecting digital impression quality [51]. It emphasized that increasing scan range and implant separation can reduce trueness, which is where enhanced scan body geometry—like extensional structures—can act as a corrective measure by improving scanner engagement and spatial referencing [51]. Our findings not only align with but also extend the current literature by providing focused evidence that geometric enhancements to scan bodies offer tangible accuracy benefits, particularly in scenarios where scan range or implant configuration poses intrinsic challenges.

Distinct macro-shape geometries—such as cuboidal versus dome-shaped designs—affected surface and centroid deviations while preserving comparable angular alignment. In studies that directly compared geometric forms, such as the article by Moslemion et al., we only included the straight scan body data to allow for a focused comparison of geometric shapes while controlling for orientation variables [24]. Our findings showed that printed scan bodies had variability in trueness, with differences between printing methods. It is in accordance with prior evidence that showed printed scan body geometry and surface topography affected digital impression accuracy [52]. Geometric features such as sharp edges, complex contours, and deep or angular surfaces can impair scanning accuracy by disrupting the generation of precise point clouds [52]. Extensional structures have been found to increase accuracy by providing more reliable reference points for the stitching algorithms used during intraoral scanning [22]. Subtractive modifications to printed scan body design have been shown to improve trueness, while additive alterations often introduce irregularities that diminish scanning precision [41]. Therefore, resin-printed scan bodies, which typically rely on layered additive techniques with lower resolution and higher surface irregularity, may be more susceptible to geometric distortion than those produced via laser sintering or milling methods.

Cylindrical scan bodies optimized for specific heights and diameters achieved the lowest 3D and angular deviations among various geometric configurations. Across several studies, cylindrical configurations—especially those tailored to platform dimensions—had an optimal fit and minimal error. This was supported by data extracted from Tan et al., where we focused only on intraoral scan bodies (excluding the laboratory designs) to ensure consistency in clinical relevance and scanning conditions [8]. The reduced error associated with these forms suggests a critical intersection between geometry and platform compatibility. The superior performance of cylindrical geometries can be attributed to their continuous, smooth curvature, which promotes uniform light reflection and reduces both specular glare and shadowing that occur at sharp edges or facets. Moreover, simplified designs with fewer facets and minimal sharp angles demonstrated superior mesh-to-library congruence and overall impression accuracy. A comprehensive systematic review also identified geometry as one of the five major factors influencing scan accuracy, though no consensus on an ideal design was found [53]. Evidence showed that overly complex geometries may lead to scanning inconsistencies, mesh distortion, or software misalignment. This is also supported by a systematic review that classified implant scan body geometry as a main operator-related source of scanning error, alongside mesh holes, stitching failures, and scanning noise—underscoring the clinical advantage of simplified, cylindrical designs [54]. In the article by Revilla-Leon and colleagues, three different scan body designs were compared, but we excluded the third group (dynamic abutment IOS) due to its incompatibility with coordinate measuring machine evaluation, as also noted by the study authors [26].

Accessory parts and geometric attachments affixed to scan bodies reduced 3D and linear deviations without compromising angular precision. These enhancements seem to provide stability and better scanner recognition while maintaining orientation fidelity. In Farah et al., when analyzing the impact of geometric attachments, we chose data from the parallel implant group to isolate attachment effects without angulation confounds [47]. Similarly, a recent systematic review evaluated application methods of scan bodies and found that attaching auxiliary geometric devices significantly improved scanning accuracy in edentulous arches, although splinting scan bodies yielded inconsistent benefits [49]. In line with these findings, Shetty et al. reported that splinting scan bodies—using techniques such as light-cured resin, pattern resin, dental floss, and custom splints—generally enhances accuracy in complete-arch digital impressions by stabilizing reference points, though effectiveness varies by clinical scenario, scanning protocol, and IOS type [55]. Such strategies highlight the functional synergy between auxiliary components and baseline scan body architecture. Manufacturer-specific design variations had differences in accuracy metrics, with certain proprietary bodies (e.g., ELOS A/S, TeamZiereis) consistently showing better performance than others (e.g., NT-Trading). This variation highlights the effects of material composition, surface texture, and design standardization on scanning outcomes. Motel et al. applied both single-step and two-step scanning protocols; for consistency with the rest of our included literature, we analyzed data from the single-step strategy only [25]. This methodological alignment allowed for cleaner comparisons between manufacturer results and reinforced the importance of design standardization in commercial scan body production. Conversely, extensive geometric modifications (e.g., large proximal slots or additive material enhancements) affected both linear and angular scanning accuracy. Such excessive alterations may disrupt scan continuity, introduce scanner artifacts, or obscure key alignment planes. In the context of angulation-related outcomes in the study by Moslemion et al., we included only data from the Doowon group, as it featured scan body shapes most similar to commonly used clinical models for preserving external validity [24].

In clinical practice, clinicians should select scan bodies with extensional features, optimized cylindrical dimensions, or simple facet designs—such as bar-extended or flat-sided geometries—to improve both linear and angular trueness in everyday workflows [27,34]. Also, manufacturers should develop standardized guidelines for validating scan body designs—including minimum geometric criteria and performance thresholds under intraoral scanning conditions to ensure consistency across implant systems [23,25]. Future investigations can use uniform testing protocols, expand in vivo and multi-operator studies to assess clinical performance, and explore the cost–benefit balance of advanced geometries versus conventional designs for informing evidence-based improvements in digital implantology.

There are some limitations. Despite growing evidence on the influence of scan body geometry, most available data stem from in vitro and animal models, which fail to fully replicate clinical conditions such as saliva, patient movement, and soft tissue dynamics. Only two in vivo studies were included, limiting the generalizability of our findings. The high heterogeneity of scanning protocols, measurement techniques, and outcome definitions precluded meta-analysis. Operator variability, implant configurations (e.g., angulation, number of implants), and IOS system settings were inconsistently reported, introducing additional potential confounders. Moreover, the underlying optical and geometric mechanisms—such as light deflection and reflection differences between curved versus angular surfaces—remain insufficiently explored. Finally, no included study assessed patient-centered outcomes, such as prosthetic fit, insertion torque, or clinical ease of use, constraining insights into real-world utility and patient comfort.

Challenges and future perspectives: Despite significant advances in scan body design research, several challenges remain that must be addressed to translate in vitro findings into clinical practice. First, the predominance of in vitro and animal studies limits our understanding of performance under clinical conditions (e.g., saliva, patient movement, soft tissue dynamics). Future work should prioritize standardized in vivo investigations using consistent scanning protocols, implant configurations, and outcome definitions. Second, heterogeneity in scanner systems, measurement methods, and geometry categorizations impedes meta-analytic synthesis and direct comparisons; consensus on terminology and protocol reporting is needed. Third, the optical–geometric interactions—such as light scattering on curved versus angular surfaces—remain underexamined; mechanistic studies could inform the rational design of next-generation scan bodies. Fourth, operator variability and system settings represent potential confounders; automated or guided scanning workflows may help reduce human error. Finally, there is a paucity of patient-centered outcomes, such as prosthesis fit, insertion torque, and clinical efficiency; future research should integrate these measures to demonstrate real-world benefits. By addressing these challenges through collaborative, multidisciplinary efforts and rigorous in vivo validation, the field can move toward evidence-based scan body innovations that enhance both clinical accuracy and workflow efficiency. While our review highlights clear geometric factors that enhance digital impression accuracy, its clinical translation is constrained by the predominance of in vitro and animal studies. Accordingly, the clinical applicability of these conclusions must be interpreted with caution, and future research should focus on well-designed in vivo validation studies to confirm performance under clinical conditions, including the influence of saliva, patient movement, and soft tissue dynamics. Moreover, the potential impact of different IOS models on measurement accuracy remains underexplored. Variations in optical hardware, software algorithms, and calibration protocols between devices may lead to device-specific biases in trueness and precision. Future studies should directly compare multiple IOS systems using standardized geometries and scanning conditions to determine how the scanner model influences scan body performance.

## 5. Conclusions

Scan body geometry is a key determinant of implant impression accuracy. Rigid extensions, such as rigid bars and wings, consistently enhance both linear and angular accuracy. Macro-shapes influence surface deviation, with cuboidal designs yielding greater errors than dome-shaped forms. Among macro shapes, simplified cylindrical forms had superior trueness compared to more complex cuboidal or spherical designs. Hybrid healing-abutment systems are comparable to conventional scan bodies for single-unit scans, with one-piece designs outperforming multi-component assemblies. Simpler geometries and targeted modifications improve scan mesh alignment, while excessive alterations compromise accuracy. At the micro level, targeted extensional features and surface facets can further refine mesh alignment, whereas excessive additive modifications often degrade accuracy by introducing irregularities. Clinically, these findings advocate for scan body designs that balance clear, simplified reference geometry with strategic micro-features to optimize both trueness and precision. Future work should build on this innovation by implementing standardized in vivo protocols, evaluating patient-centered outcomes (e.g., prosthetic fit and ease of use), and exploring the optical–geometric mechanisms underpinning scanner–geometry interactions. These steps will pave the way for evidence-driven scan body development and improved digital prosthodontic care.

## Figures and Tables

**Table 1:** Characteristics of the included studies.

Study ID	Study Design	Scan Body Geometry	Sample Size	Measurement	Implants
Pan et al., 2022 [33]	In vitro	Cuboidal dome-shaped (both fabricated by the same manufacturer (ZfxTM Intrascan matchholder H4 and ZfxTM Evolution matchholder, Zimmer Biomet, Indiana, USA))	4	Iterative-closest point (ICP) criteria	Six
Motel et al., 2020 [25]	In vitro	ELOS A/S, Gørløse, Denmark NT-Trading GmbH, Karlsruhe, Germany TeamZiereis, Baden-Württemberg, Germany	10	Local best fit	Three
Huang et al., 2021 [27]	In vivo	Scan body with extensional structure (a rigid bar) scan body without extensional structure		Best-fit algorithm	Two
Huang et al., 2020 [22]	In vitro	Original scan body (Straumann, Basel, Switzerland)—CAD/CAM scan body without extensional structure—CAD/CAM scan body with extensional structure	10	Best-fit algorithm	Four
Revilla-León et al., 2020 [26]	In vitro	Elos Medtech, Gørløse, Denmark, Elos Accurate Intraoral Scan body Nt-Trading, Karlsruhe, Germany, 3D Guide Intraoral Scan body Dynamic Abutment (Talladium, Lleida, Spain), Intraoral scan body system with intraoral adaptor	10	Best-fit technique	Three
Revilla-León et al., 2021 [28]	In vitro	Elos accurate IO scanbody Brånemark system RP; Nobel Biocare Services AG Scan Body 3D Guide K Series; NT Digital Implant Technology	NA	NA	NA
Meneghetti et al., 2023 [34]	In vitro	SB1: Cylinder, with a trapezoidal in the top, with different surfaces. 14 mm high PEEK with a metal connection S.I.N., São Paulo, Brazil SB2: Cylinder with an angled flat surface, 9 mm high PEEK Neodent, Curitiba, Brazil SB3: Cylinder with diamond surfaces, 12 mm high PEEK Neodent, Curitiba, Brazil SB4: Prototype, rounded, 7 mm high 3D printed, grey resin, Custom SB5: Prototype, tri-angled flat surfaces, 7 mm high 3D printed, grey resin, Custom SB6: Prototype, bar 16 mm with a convex ball in the extremity 3D printed, grey resin, Custom SB7: Cylinder with an angled flat surface, 7 mm high PEEK S.I.N., São Paulo, Brazil	10	Using an ADD-ON for Blender called Object align/ICP align	Six
Ramadan et al., 2023 [35]	In vitro	One-piece SB (Elos Medtech) Two-piece Healing Abutment-Scan Peg (HA-SP; Neoss, Harrogate, HG1 2PW, United Kingdom)	10	Best-fit matching	One
Yilmaz et al., 2021 [30]	In vitro	Conventional intraoral scan body (CSB) (Neoss, Woodland Hills, CA, USA) A healing abutment-scanpeg system (HASP)	10	Local best-fit	One
Lawand et al., 2024 [41]	In vitro	CARES Mono Scanbody for a screw-retained abutment (nonmodified) Subtractively modified scan body Additively modified scan body	15	Reference best-fit algorithm	Two
Alvarez et al., 2022 [31]	In vitro	ELOS, MG, Ticare MG, and Talladium. Model 3a-B ELOS Medtech Denmark (ELOS), one piece, screwed in, a milled angulated side Mozo Grau S.A scan body, a milled pyramidal side, screwin placement, and two-piece clip-in system (MG) Mozo Grau S.A scan body, 12 milled sides, screw-in, one piece (Ticare MG) Talladium scan body (Talladium Spain), a milled side, magnetic placement, and 2 pieces	10	Best-fit	Six
Mizumoto et al., 2019 [23]	In vitro	AF (IO-Flo; Dentsply Sirona, 63457 Hanau, Germany) NT (Nt-Trading GmbH & Co KG, Karlsruhe, Baden-Württemberg, Germany) DE (DESS-USA, Lake Mary, FL 32746, USA) C3D (Core3Dcentres, Castle Hill, Australia) ZI (Zimmer Biomet Dental, Palm Beach Gardens, Florida 33410, USA)	5	Best-fit algorithm	Four
Jung et al., 2022 [32]	In vitro	Simple scan abutment (IHAB 50 06 H, Dentium, Gyeonggi-do, Republic of Korea) Scanning jig (SCJ I4565, Dentium, Gyeonggi-do, Republic of Korea)	10	Specific anatomic structures in the teeth adjacent to the implants were set as reference points, and the mean 3D linear intra-arch and interarch deviations were evaluated	Two
Moslemion et al., 2020 [24]	In vitro	DESS 14.005 NT-Trading E9.S3D4.300 Doowon B051	10	Best-fit algorithm	Four
Schmidt et al., 2021 [29]	In vitro	3D Guide, H-Series, NT Trading, Karlsruhe, Germany Cara H10/20, Kulzer, Hanau, Germany H1410, Medentika, Niefern-Öschelbronn, Germany	10	Reference system, which allowed determination of the exact x-, y-, and z-deviations	Four
Tan et al., 2022 [8]	In vitro	Medentika L-Series Scan body Second Generation (REF L1420) (Medentika) Straumann CARES Mono Scan body for implant-level scanning (REF 025.4915) (SM) Core 3D Scanbody, Straumann Bone level RC (compatible) (REF 2077) (C3D) Straumann RC Scan body (REF 025.4905) (SS)	10	Zero method	Ten
Li Y. et al., 2024 [42]	In vitro	Conventional scan bodies (CARES Mono Scanbody; Institute Straumann AG for IOS-C, Basel, Switzerland) Modified scan bodies (Digital Wings; Segma Medical Technology for IOSM, Beijing, China)	10	Best-fit algorithm	Six
Zhang et al., 2024 [45]	In vitro	Original scan bodies (group OS) Computer-aided design and computer-aided manufacturing (CAD/CAM) scan bodies without extension (group CS) CAD/CAM scan bodies with straight extension (group CSS) CAD/CAM scan bodies with arcuate extension (group CSA)	10	Best-fit algorithm	Four
Alkindi et al., 2024 [38]	In vitro	Short scan bodies (SSB) Long scan bodies (LSB)	10	Best-fit algorithm	Two
Park et al., 2024 [44]	In vitro	Conventional scan bodies with no vertical stop (nS) Experimental scan bodies with a vertical stop (S)	10	A computerized coordinate measuring machine (CMM: Contura; Zeiss) was used to probe the reference casts, and a computational software program (Calypso; Zeiss) was used to compute the 3D coordinates of the implant platform centroids and projection angles of the implant long axes.	Three
Pan et al., 2025 [48]	In vitro	Nine cylinders (⌀4.8 × 4 mm, ⌀4.8 × 8 mm, ⌀4.8 × 12 mm, ⌀5.5 × 4 mm, ⌀5.5 × 8 mm, ⌀5.5 × 12 mm, ⌀6.5 × 4 mm, ⌀6.5 × 8 mm, ⌀6.5 × 12 mm) Five cuboids (3 × 6 × 8 mm, 3 × 6 × 12 mm, 4 × 6 × 6 mm, 5 × 6 × 12 mm, 5 × 6 × 8 mm) Sphere (⌀8 mm)	7	Direct measurements are based on a physical standard reference point, which serves as the ground truth with established Cartesian coordinates, without resorting to virtual alignment procedures.	NA
Ashry et al., 2024 [40]	In vitro	Scan bodies without accessory parts—scan bodies with accessory parts	20	Best-fit algorithm	Four
Farah et al., 2025 [47]	In vitro	With geometrical attachments Without geometrical attachments	20 intraoral scans (10 per scanner: 5 with geometric attachments, 5 without)	best-fit algorithm	Four
Michelinakis et al., 2024 [43]	In vitro	Straumann Cares Mono RN (STR) Paltop SP (PLT)—MIS SP V3 (MIS) TRI TV70 scan (TRI)	10	Best-fit algorithm	Four
Uzel et al., 2023 [37]	In vitro	Group 1: No modifications Group 2: A 2 mm × 3 mm slot was made on the proximal surface without damage to the occlusal surface Group 3: A 3 mm × 4 mm slot was made on the proximal surface without damage to the occlusal surface Group 4: A 3 mm × 6 mm slot was made including the occlusal and proximal surfaces	10	Best-fit registration program	Two
Shely et al., 2023 [36]	In vitro	MIS ISB (asymmetrical geometry, trapezoid with sharp angles with a larger surface area compared to the ZZ ISB, internal hex connection) (MIS) Zirkonzhan ISB (cylindrical with no angles/asymmetric geometry, internal hex connection) (ZZ)	30	Best-fit algorithm	Three
Eldabe et al., 2025 [46]	In vivo	Tooth-modified scan body (TMSB) Conventional scan body (CSB)	total sample size: 68 (2 scans per implant)	“N-point registration” and “global registration” algorithms	4 (n = 1) and 5 implants (n = 6)
Anwar et al., 2024 [39]	In vitro	Implant level scan bodies (CopaSky; bredent medical, Senden, Germany) (NM) Modified scan bodies (round depressions were ground into the center of the buccal and palatal surfaces of the scan bodies without interfering with the coronal geometric bevel of the SB) (M)	10	Best-fit algorithm	Four

Abbreviations: NA: not available; ICP: iterative closest point; CMM: coordinate measuring machine; SCJ: scanning jig; SB: scan body; HA-SP: healing abutment–scan peg; CSB: conventional scan body; IOS: intraoral scanner.

**Table 2:** Summary of scan body geometries and their impact on accuracy.

Geometry Type	Reported Impact on Accuracy	Key References
Cylindrical (various heights/diameters)	Best linear and angular trueness, especially with 5.5 mm diameter and 12 mm height	[8,48]
Cuboidal	Generally worse trueness and angular accuracy; affected by height and cross-section area	[48]
Spherical	Intermediate trueness; angular accuracy not measurable due to shape	[48]
With rigid bar extension	Improved trueness and angular deviation in both in vitro and in vivo settings	[27]
With extensional structure	Reduced mean deviation from ~119 µm to ~69 µm and angular error from ~0.75° to ~0.36°	[22]
Subtractive modification	Improved angular accuracy vs. non-modified and additive types	[41]
Additive modification	Degraded surface trueness; introduced irregularities	[41]
With surface facets (diamond, trapezoid, etc.)	Mixed impact; some improved trueness, others not significant	[26,34]
Healing abutment-scan peg (hybrid)	Comparable or slightly worse than one-piece bodies	[30,35]
Tooth-modified scan body	Significantly reduced 3D deviations and angular errors	[46]
Depression-type scan body	Improved surface trueness (from ~0.282 mm to ~0.229 mm)	[39]

## Data Availability

The data that supports the findings of this study are available in the Supplementary Materials of this article.

## Supplementary Materials

The following supporting information can be downloaded at https://www.mdpi.com/article/10.3390/dj13060252/s1, Table S1: Search strategy for PubMed, Scopus, Embase, Web of Science, and Cochrane library databases. Table S2. Results and outcomes of the included studies. Table S3: Results of the quality assessment of the included studies.

## Abbreviations

The following abbreviations are used in this manuscript:

IOSs	Intraoral scanners
PICO	Population, intervention, comparison, outcome
PRISMA	Preferred Reporting Items for Systematic Reviews and Meta-Analyses
MeSH	Medical Subject Headings
CAD/CAM	Computer-aided design/computer-aided manufacturing
QUIN	Quality Assessment Tool For In Vitro Studies
PEEK	Polyether ether ketone
RMS	Root mean square
