# Supplementary material for: The Effects of Scan Body Geometry on the Precision and the Trueness of Implant Impressions Using Intraoral Scanners: A Systematic Review
Source: Dent J (Basel). 2025 Jun 5;13(6):252. doi: 10.3390/dj13060252 (PMC12191632; doi:10.3390/dj13060252)
Supplement: Supplementary file 1 [file dentistry-13-00252-s001.zip › dentistry-3638557-supplementary/V2 Supplementary Material, Geometry.docx]

**­Table S1.** Search strategy for PubMed, Scopus, Embase, Web of Science, and Cochrane library databases.

| **Step** | **Query** | **No. of results** |
| --- | --- | --- |
| **PubMed** | | |
| 1 | "Dental Implants"[MeSH Terms] OR "Dental Implantation"[MeSH Terms] OR "dental implantation, endosseous, endodontic"[MeSH Terms] OR "dental implantation, endosseous"[MeSH Terms] OR "Dental Implant-Abutment Design"[MeSH Terms] OR "dental prosthesis, implant supported"[MeSH Terms] OR "dental implants, single tooth"[MeSH Terms] OR "Prostheses and Implants"[MeSH Terms] OR "Bone-Anchored Prosthesis"[MeSH Terms] | 625,499 |
| 2 | "scan bod*"[Title/Abstract] OR "scan abutment*"[Title/Abstract] OR "scanbod*"[Title/Abstract] OR "scan post*"[Title/Abstract] OR "scanpost*"[Title/Abstract] | 481 |
| 3 | "Data Accuracy"[MeSH Terms] OR "Dimensional Measurement Accuracy"[MeSH Terms] OR "Reproducibility of Results"[MeSH Terms] OR "accuracy"[Title/Abstract] OR "trueness"[Title/Abstract] OR "validity"[Title/Abstract] OR "precision"[Title/Abstract] OR "reliability"[Title/Abstract] OR "measured deviation*"[Title/Abstract] OR "reproducibility"[Title/Abstract] | 1,484,150 |
| 3 | #1 AND #2 AND #3 | 282 |
| **Scopus** | | |
| 1 | (TITLE-ABS-KEY("Dental Implant-Abutment Design" OR "dental prosthesis, implant supported" OR "dental implants, single tooth" OR "Prostheses and Implants" OR "Bone-Anchored Prosthesis" OR "Dental Implants" OR "Dental Implantation" OR "dental implantation, endosseous, endodontic" OR "dental implantation, endosseous")) AND (TITLE-ABS-KEY("scan body" OR "scan bodies" OR "scanbody" OR "scanbodies" OR "scan post" OR "scan posts" OR "scanpost" OR "scanposts" OR "scan abutment" OR "scan abutments")) | 290 |
| **Embase** | | |
| 1 | ('dental implant':ti,ab OR 'dental implants':ti,ab OR 'dental implantation':ti,ab OR 'dental implantation, endosseous':ti,ab OR 'dental implantation, endosseous, endodontic':ti,ab OR 'dental implantation, subperiosteal':ti,ab OR 'endodontic endosseous dental implantation':ti,ab OR 'endosseous dental implantation':ti,ab OR 'dental implants, single-tooth':ti,ab OR 'single dental implant':ti,ab OR 'single tooth implant':ti,ab OR 'single-tooth dental implants':ti,ab) AND (scanbody:ti,ab OR 'scan body':ti,ab OR scanbodies:ti,ab OR 'scan bodies':ti,ab OR scanpost:ti,ab OR 'scan post':ti,ab OR scanposts:ti,ab OR 'scan posts':ti,ab OR 'scan abutment':ti,ab OR 'scan abutments':ti,ab) | 51 |
| **Web of Science** | | |
| 1 | TS=(dental implant*) | 227 |
| 2 | ((((TS=("scan bod*")) OR TS=(scanbod*)) OR TS=("scan post*")) OR TS=("scanpost*")) OR TS=("scan abutment*") |  |
| 3 | #1 AND #2 |  |
| **Cochrane library** | | |
| 1 | MeSH descriptor: [Dental Implants] explode all trees | 7 |
| 2 | MeSH descriptor: [Dental Implantation] explode all trees |  |
| 3 | “dental implant” |  |
| 4 | #1 OR #2 OR #3 |  |
| 5 | "scan body" OR "scanbody" OR "scanbodies" OR "scan bodies" OR "scanpost" OR "scanposts" OR "scan post" OR "scan posts" OR "scan abutment" OR "scan abutments" |  |
| 6 | #4 AND #5 |  |

**Table S2.** Results and outcomes of the included studies.

| **Study ID** | **Linear Displacement** | **Angular Displacement** | **Analysis** |
| --- | --- | --- | --- |
| Pan et al. 2022 [1] | Mean ± SD of linear, µm: deviations of the model surface: dome-shaped: mean 10.7 ± 0.2 cuboidal group: mean 13.9 ± 0.7 Linear deviation of scan body centroid(µm/median [IQR]): Cuboidal : 14.6 (9.1–27.5) Dome-shaped: 12.2 (11.4–19.0) p=0.495 | Angular deviation of scan body Centre axis (°/median [IQR]): Cuboidal: 0.088 (0.030–0.108)  Dome-shaped: 0.085 (0.068–0.108)  p=0.091 | R difference:  The cuboidal group showed significant greater deviations of the model surface than the dome-shaped group: p < .05  Ɵ difference: No significant differences in angular displacement of the center-axes of the scan body were found between 2 groups (p = .091). |
| Motel et al. 2020 [2] | Mean deviation(mm): ELOS A/S: 0.041 (lowest deviation) ELOS A/S: X=0.015, Z=0.015  NT-Trading: 0.112(highest deviation) NT-Tradingl: X=0.076 mm, Z=0.040 TeamZiereis: X=0.035, Z=0.014 No significant differences in accuracy were found concerning the y-axis | NA | R difference: p = 0.003 ELOS A/S less displacement than NT-Trading P=0.003 TeamZiereis less displacement than NT-Trading P=0.022 |
| Huang et al. 2021 [3] | Mean ± SD of linear (µm): Scan body without extensional structure: 119.53 ± 83.27 Scan body with extensional structure: 68.89 ± 31.34 | Mean ± SD of angular (degree): Scan body without extensional structure: 0.75 ± 0.79 Scan body with extensional structure: 0.36 ± 0.29 | R difference: Scan body with extensional structure was more accurate than scan body without extensional structure (p=0.008). Ɵ difference: Scan body with extensional structure was more accurate than scan body without extensional structure(p=0.049). |
| Huang et al. 2020 [4] | The median (IQR) of trueness(μm): Original scan body: 35.85 (29.80-49.10)  CAD/CAM scan body without extensional structure: 38.50 (35.35-52.58) CAD/CAM scan body with extensional structure: 28.45 (24.88-36.43) | NA | Significant differences for trueness (P = 0.001) Pairwise comparison: No significant differences between original scan body and CAD/CAM scan body without extensional structure (P = 1.000), Original scan body and CAD/CAM scan body with extensional structure (P = 0.461), CAD/CAM scan body without extensional structure and CAD/CAM scan body with extensional structure (P = 0.133). |
| Revilla-León et al. 2020 [5] | 3D linear discrepancy(mm): Elos Medtech: 4 ±100 Nt-Trading: 8 ±52 Dynamic Abutment: 5 ±19 | NA | R difference: No significant differences were found in the x-, y-, and z-axes between the different groups. Ɵ difference: XZ Ɵ difference: Nt-Trading group was significantly lower than that of the Elos Medtech and Dynamic Abutment groups. YZ Ɵ difference: Dynamic Abutment was significantly higher than that for Nt-Trading groups. |
| Revilla-León et al. 2021 [6] | Median ± IQR (µm): Elos accurate IO scanbody: x-Axis: -18.8 ±95.2 y-Axis: 2.6 ±95.3 z-Axis: 0 ±0 Scan Body 3D Guide K Series: x-Axis: 11.4 ±66.9 y-Axis: 1.9 ±79.5 z-Axis: 0 ±0 | Median ± IQR (degrees): Elos accurate IO scanbody: XZ Angle: 0.5 ±0.2 YZ Angle: 0.0 ±0.5 Scan Body 3D Guide K Series: XZ Angle: -0.0 ±0.4 YZ Angle: 0.0 ±0.2 | R difference: No significant differences were found between the different groups.  Ɵ difference: YZ Ɵ difference: No significant differences were observed between the groups. Elos accurate IO scanbody group demonstrated a significantly higher XZ Ɵ difference than the Scan Body 3D Guide K Series group (P<.001). |
| Meneghetti et al. 2023 [7] | 3D deviation in median (μm): SB1: 118.47 SB2: 72.27 SB3: 158.23 SB4: 165.08 SB5: 190.29 SB6: 182.51 SB7: 93.31 | angular (˚) deviation in median: SB1: 0.45 SB2: 0.25 SB3: 0.63 SB4: 0.77 SB5: 0.89 SB6: 0.71 SB7: 0.51 | R difference: Significant differences between SBs (p<0.001). The lowest median values for 3D deviation were obtained by SB2. Ɵ difference: Significant differences in the SBs (p<0.001) |
| Ramadan et al. 2023 [8] | Mean±SD: 3D linear deviation (μm): Elos Medtech: 0.054±0.001 HA-SP: 0.182±0.004 | Mean±SD: Angular deviation in vertical direction (°):  Elos Medtech: 0.379±0.023 HA-SP: 1.676±0.073  Angular deviation in horizontal direction (°):  Elos Medtech: 0.288±0.071 HA-SP: 0.571±0.044 | R difference: significant differences  Ɵ difference: significant differences  Elos Medtech group showed statistically significant less deviation values compared to HA-SP group (P < 0.001). Elos Medtech group had higher trueness values than HA-SP group. |
| Yilmaz et al. 2021 [9] | Mean distance [mm] ± standard deviations (SD) CSB: Point 1: 0.014 (±0.015) Point 2: 0.031 (±0.033) Point 3: 0.043 (±0.041) Point 4: 0.043 (±0.044) HASP: Point 1: 0.076 (±0.013)  Point 2: 0.05 (±0.044)  Point 3: 0.178 (±0.059)  Point 4: 0.094 (±0.081) 1 – implant abutment-connection, 2- most buccal-coronal, 3 - middle point on buccal coronal slope, 4 -most palatal coronal point | Mean angular [degree] ± standard deviations (SD) CSB: buccopalatal: 0.208 (±0.237)  mesiodistal: 0.273 (±0.205) HASP: buccopalatal: 0.195 (±0.193) mesiodistal: 0.186 (±0.239) 1 - implant-abutment-connection, 2 - most buccal-coronal, 3 –middle point on buccal coronal slope, 4 – most palatal point on top of scanpeg | R difference and Ɵ difference: not significant |
| Lawand et al. 2024 [10] | Overall RMS, Mean ±SD (mm): Nonmodified: 0.092 ±0.015 Subtractively modified: 0.266 ±0.030 Additively modified: 0.100 ±0.004 | Global angular deviation, Mean ±SD (Degrees): Nonmodified: 1.266 ±0.074 Subtractively modified: 1.273 ±0.120 Additively modified: 0.993 ±0.062 | R difference:  The additively modified group presented the highest mean 3D surface discrepancies compared with the subtractively modified and nonmodified groups (P<.001). No significant 3D surface discrepancies were found between the subtractively modified and nonmodified groups (P>.999). Ɵ difference:  Subtractively modified also had the lowest mean angular deviation value compared with nonmodified and additively modified,with no significant difference between nonmodified and additively modified (P>.999). |
| Alvarez et al. 2022 [11] | Distance errors, Mean ±SD (mm): ELOS: 0.083 ± 0.072 MG: 0.082 ± 0.065 Ticare MG: 0.050 ± 0.039 Talladium: 0.041 ± 0.024 | Errors in angulation, Mean ±SD (Degrees): ELOS: 0.836 ± 0.804 MG: 0.371 ± 0.272 Ticare MG: 0.185 ± 0.189 Talladium: 0.221 ± 0.186 | R difference:  Significant differences were found of ELOS with Ticare MG and Talladium (P<.01).  Ɵ difference:  Significant differences were found between ELOS and MG, and also between Ticare MG and Talladium (P<.01). Ticare MG Scan Bodies and the Talladium are more accurate for the three parameters than the other two(ELOS and MG). The main average differences found were: - In distance, between ELOS and Talladium, with Flat side(one) providing better results (0.041 ± 0.013 mm CI 95%). - In angulation: between ELOS and Ticare MG, with the latter being more accurate (0.644 ± 0.143 degrees IC 95%). |
| Mizumoto et al. 2019 [12] | NA | C3D-NT: difference =-0.79  DESS-NT: difference =0.73 | R difference: P=0.031 ZI showed significantly less distance deviation than AF (P=.041) Ɵ difference: P<0.001 pairwise comparison: C3D-NT: p=0.009 DESS-NT: p=0.023 |
| Jung et al. 2022 [13] | Mean total linear intra-arch deviation was less than 100 μm in both groups. Mean total linear inter-arch deviation was less than 100 μm in both groups. | Scanning jig(Dentium): Second premolar: Ɵ=1.17 ±0.52  First molar: Ɵ=1.05 ±0.61 Simple scan abutment(Dentium): Second premolar: Ɵ=1.38 ±0.35  First molar: Ɵ=1.41 ±0.48 | Implant position: P>0.05 Ɵ difference: P>0.05 Simple scan abutment higher intra-arch deviation than scanning jig (P<.05). |
| Moslemion et al. 2020 [14] | Mean±Standard Deviations: DESS: 0.17±0.07(mm)  NT-Trading: 0.06±0.02  Doowon: 0.05±0.03 | Mean±Standard Deviations: DESS: Ɵ=0.47±0.3  NT-Trading: Ɵ=0.35±0.19  Doowon: Ɵ=0.52±0.21 | R difference : p <0.001 DESS had more R difference compared to the other groups (p <0.001) |
| Schmidt et al. 2021 [15] | Mean(Trueness) ± SD(Precision),mm:  Implant Position 14:  NT: 0.134±0.026 (mm) Kulzer: 0.114±0.037 Medentika: 0.106±0.050  Implant Position 16: NT: 0.164±0.032 Kulzer: 0.161±0.045 Medentika: 0.123±0.041  Implant Position 24: NT: 0.180±0.034 Kulzer: 0.175±0.075 Medentika: 0.191±0.052  Implant Position 26: NT: 0.183±0.038 Kulzer: 0.199±0.066 Medentika: 0.197±0.052 | NA | No significant differences could be found in terms of trueness between different scanbodies. |
| Tan et al. 2022 [16] | Mean global linear distortions(μm): Medentika: 17.8 ±6.8 SM: 11.4 ±6.3 C3D: 32.5 ±17.5 SS: 42.5 ±23.5 | NA | R difference: P<0.001 |
| Li Y. et al. 2024 [17] | RMS: Observed maximum discrepancy for the modified scan bodies: 37.5 μm | NA | Modified scan bodies had better trueness compared with that of conventional group (P<.001) |
| Zhang et al. 2024 [18] | Median trueness, μm: OS: 41.40, CS: 55.95, CSS: 39.80, CSA: 39.75 | NA | Significant differences in trueness was found among the groups. (P<.001)  There were no statistically significant differences between CSA and OS (P=1.000), CS (P=.050), CSS (P=1.000), and CI (P=.132). No significant differences in trueness were found between OS and CS (P=.277) OS and CSS (P=1.000), and CS and CSS (P=.182). |
| Alkindi et al. 2024 [19] | The trueness of the platform deviation, μm: SSB: range= 37.1 to 51.9 LSB: range= 89.6 to 127.9 | The trueness of the angle deviation, degrees: SSB: range=0.11 to 0.25 degrees  LSB: range= 0.31 to 0.57 degrees | R difference: The trueness of SSB was significantly better than LSB (p<0.001). Ɵ difference: The trueness of the angle deviation of digital scans with SSB was significantly better than scans with LSB. |
| Park et al. 2024 [20] | 3D deviations: First premolar: p<.001 7-degree internal conical angle: nS: 0.048 ±0.011   S:0.030 ±0.009 11-degree internal conical angle: nS:0.182 ±0.061   S: 0.129 ±0.040 First molar: p<.001 7-degree internal conical angle: nS: 0.046 ±0.014  S: 0.055 ±0.011 11-degree internal conical angle: nS:0.171 ±0.039  S: 0.131 ±0.061 Second molar: p<.001 7-degree internal conical angle: nS: 0.039 ±0.014  S: 0.058 ±0.017 11-degree internal conical angle: nS: 0.145 ±0.037  S: 0.101 ±0.028 All: p <.001 7-degree internal conical angle: nS: 0.039 ±0.014  S: 0.120 ±0.046 11-degree internal conical angle: nS: 0.044 ±0.013  S: 0.048 ±0.018 | First premolar: ΔθZX: 7-degree internal conical angle: nS: -0.642 ±0.106  S: -0.857 ±0.095 11-degree internal conical angle: nS: -1.210 ±0.107  S: -1.216 ±0.101 p<.001 ΔθYZ: 7-degree internal conical angle: nS: 0.423 ±0.090  S: 0.430 ±0.058 11-degree internal conical angle: nS: 0.364 ±0.344  S: 0.091 ±0.180 p=.001 ΔθXY: 7-degree internal conical angle: nS: -0.368 ±0.092  S: -0.352 ±0.057 11-degree internal conical angle: nS: -0.464 ±0.340  S: -0.198 ±0.179 p=.041 First molar: ΔθZX: 7-degree internal conical angle: nS: 0.034 ±0.090  S: 0.020 ±0.048 11-degree internal conical angle: nS: 0.128 ±0.154  S: 0.010 ±0.245 p=.309 ΔθYZ: 7-degree internal conical angle: nS: 0.500 ±0.154  S: 0.402 ±0.106 11-degree internal conical angle: nS: 0.219 ±0.204  S: 0.252 ±0.293 p=.011 ΔθXY: 7-degree internal conical angle: nS: -0.503 ±0.153  S: -0.403 ±0.109 11-degree internal conical angle: nS: -0.158 ±0.194   S: -0.137 ±0.311 p<.001 Second molar: ΔθZX: 7-degree internal conical angle: nS: 1.320 ±0.131  S: 1.359 ±0.074 11-degree internal conical angle: nS: -0.240 ±0.236  S: -0.196 ±0.299 p<.001 ΔθYZ: 7-degree internal conical angle: nS: 0.793 ±0.111  S: 0.582 ±0.112 11-degree internal conical angle: nS: 0.202 ±0.221  S: 0.102 ±0.198 p<.001 ΔθXY: 7-degree internal conical angle: nS: -0.968 ±0.118  S: -0.795 ±0.124 11-degree internal conical angle: nS: -1.491 ±0.227   S: -1.390 ±0.213 p<.001 All: ΔθZX: 7-degree internal conical angle: nS: 0.237 ±0.835  S: 0.174 ±0.930 11-degree internal conical angle: nS: -0.441 ±0.598  S: -0.468 ±0.589 p<.001 ΔθYZ: 7-degree internal conical angle: nS: 0.572 ±0.200  S: 0.471 ±0.122 11-degree internal conical angle: nS: 0.262 ±0.265  S: 0.149 ±0.233 p<.001 ΔθXY: 7-degree internal conical angle: nS: -0.613 ±0.287  S: -0.517 ±0.224 11-degree internal conical angle: nS: -0.704 ±0.632  S: -0.575 ±0.631 p=.506 | R difference: Significant differences were noted in all linear displacement variables among the 4 digital cast groups, except for Δx in the left first premolar implant. For the 7-degree ICCI, the linear displacement was statistically similar in the experimental and conventional scan bodies. However, for the 11-degree ICCIs, the experimental scan body group resulted in significantly smaller Δy, Δz, and Δd (Δd2=Δx2+Δy2+Δz2) than the conventional scan body group (P<.05). Ɵ difference: Significant differences were noted in angular displacements among the test groups (P<.05), except for ΔθZX in first molar and All ΔθXY. However, there was no consistent displacement trend according to implant or scan body type. |
| Pan et al. 2025 [21] | Linear trueness, μm: Cylinder:  4.8✕12: 20.9±10.3 5.5✕12: 11.0±9.1  6.5✕12: 6.5±5.1  4.8✕8: 10.8±6.7  5.5✕8: 6.1±4.5  6.5✕8: 7.5±4.8  4.8✕4: 9.1±5.3  5.5✕4: 4.0±2.4  6.5✕4: 10.9±4.6  Cuboid: 4✕6✕6: 28.8±8.6  5✕6✕8: 15.3±6.6  3✕6✕8: 18.7±8.8  3✕6✕12: 11.9±7.2  5✕6✕12: 13.7±7.2  Sphere:  ⌀8: 12.5±8.9 | Angular trueness: Cylinder:  4.8✕12: 0.022±0.010 5.5✕12: 0.013±0.010 6.5✕12: 0.034±0.019 4.8✕8: 0.060±0.020 5.5✕8: 0.037±0.020 6.5✕8: 0.122±0.078 4.8✕4: 0.158±0.061 5.5✕4: 0.056±0.045 6.5✕4: 0.094±0.064 Cuboid: 4✕6✕6: 0.178±0.010 5✕6✕8: 0.032±0.014 3✕6✕8: 0.013±0.009 3✕6✕12: 0.022±0.138 5✕6✕12: 0.020±0.006 Sphere:  ⌀8: As spheres lack a centre-axis, angular trueness was not determined. | R difference: Best linear accuracy: Cylinder (⌀5.5 × 4 mm)  Linear trueness affected by: Height (p = 0.034) Diameter (p = 0.001) Interaction (p = 0.007)  Worst trueness: Cuboid (4 × 6 × 6 mm)  Linear trueness comparison: Cylinder: 9.5 μm (significantly better) Cuboid: 17.7 μm (p < 0.001) Sphere: 12.5 μm (p < 0.001)  Ɵ difference: Best angular trueness: Cylinder (⌀5.5 × 12 mm) Cuboid (3 × 6 × 8 mm)  Worst angular trueness: Cuboid (4 × 6 × 6 mm)   Angular trueness comparison: Cylinder: 0.065° Cuboid: 0.050° (significantly better, p < 0.001) Cylindrical group influences:  Angular trueness affected by: Height (p < 0.001) Diameter (p < 0.001) Interaction (p = 0.004)  Scan body height effect (cylindrical group):  12 mm height: Linear accuracy: 12.8 ± 10.2 μm (p = 0.056) Angular accuracy: 0.016 ± 0.010° (p < 0.001) (better than 4 mm) 4 mm height: Trueness: 7.7 ± 5.0 μm / 0.103 ± 0.070° Scan body diameter effect:  Best trueness: ⌀5.5 mm Linear: 7.1 ± 6.5 μm Angular: 0.035 ± 0.033° Worst trueness: ⌀4.8 mm Linear: 13.3 ± 9.2 μm Angular: 0.080 ± 0.070° Cuboidal group influences:  Linear trueness: No significant effect of: Height (p = 0.092) Cross-sectional area (p = 0.747) Interaction (p = 0.285) Angular trueness: 24 mm²: 0.178 ± 0.010° (significantly worse) 18 mm²: 0.018 ± 0.012° (p < 0.001) 30 mm²: 0.027 ± 0.012° (p < 0.001) |
| Ashry et al. 2024 [22] | Trueness of 3D surface deviations (mm): Mean ± SD site of the implants: SB1 at 17, SB2 at 13, SB3 at 22, and SB4 at 27 Scan bodies without accessory parts: SB1: 0.166 ±0.079 SB2: 0.182 ±0.069 SB3: 0.123 ±0.095 SB4: 0.371 ±0.169 Overall: 0.210 ±0.058 Scan bodies with accessory parts: SB1: 0.163 ±0.069  SB2: 0.157 ±0.048  SB3: 0.122 ±0.078  SB4: 0.279 ±0.094  Overall: 0.180 ±0.039 | Mean values of angular deviations (degrees): Mean ± SD site of the implants: SB1 at 17, SB2 at 13, SB3 at 22, and SB4 at 27 Scan bodies without accessory parts: SB1: 1.555 ± 0.356  SB2: 0.572±0.170  SB3: 1.104± 0.239  SB4: 0.939± 0.429  Overall: 1.043± 0.193  Scan bodies with accessory parts: SB1:1.649 ±0.506 SB2: 0.552 ±0.253  SB3: 1.226 ±0.300  SB4: 0.947 ±0.372  Overall: 1.093 ±0.183 | R difference: Scan body accessories significantly reduced 3D and linear deviations, particularly at SB4 for 3D deviation (p = 0.043) Ɵ difference: No significant effect of scan body accessories on angular deviation |
| Farah et al. 2025 [23] | RMS error (3D Mean ± SD, μm): OmniCam Without: 70.8 ± 10.3 With: 35.2 ± 3.6  iTero: Without: 47.2 ± 8.0 With: 19.6 ± 3.0 | NA | R difference: Geometric attachments significantly improved scan accuracy (p < 0.001) |
| Michelinakis et al. 2024 [24] | Mean ± SD, mm: MIS: 0.019 0.007 STR: 0.019 0.007 TRI: 0.029 0.009 PLT: 0.046 0.007 | NA | R difference: STR and MIS were significantly more accurate compared with PLT and TRI. TRI was significantly more accurate compared with PLT. PLT was significantly less accurate compared with the rest of the ISBs (P<.05) A design with a less complex shape and fewer sharp line angles and a design with a cylindrical shape exhibited statistically significantly higher congruence between the clinical mesh and the software library files. |
| Uzel et al. 2023 [25] | Linear discrepancies, mm: Group 1: Itero: 23.8 ± 13.4   3Shape: 14.9 ± 5.4   Medit: 20.3 ± 7.5   Overall: 19.6 ± 9.9 Group 2: Itero: 28.1 ± 14.6   3Shape: 16.5 ± 5.7   Medit: 21.8 ± 10.2   Overall:19.6 ± 9.9  Group 3: Itero: 51.2 ± 20.7   3Shape: 32.1 ± 8.5   Medit: 40.6 ± 15.3   Overall: 19.6 ± 9.9  Group 4: Itero: 137.5 ± 41.7   3Shape: 74.1 ± 28.2   Medit: 100.8 ± 40.3  Overall: 19.6 ± 9.9 | Angular discrepancies, mm: Group 1: Itero: 0.13 ± 0.04   3Shape: 0.17 ± 0.08   Medit: 0.19 ± 0.09   Overall: 0.16 ± 0.11 Group 2: Itero: 0.29 ± 0.2  3Shape: 0.28 ± 0.15   Medit: 0.32 ± 0.21  Overall: 0.29 ± 0.18 Group 3: Itero: 0.69 ± 0.35  3Shape: 0.71 ± 0.32   Medit: 0.68 ± 0.34   Overall: 0.69 ± 0.32  Group 4:Itero: 2.56 ± 1.88   3Shape: 1.08 ± 0.48   Medit: 1.43 ± 0.57   Overall: 1.69 ± 1.31 | R difference: The overall linear discrepancies: statistical significances (P < 0.05) among the groups except Groups 1 and 2.  Ɵ difference: The overall angular discrepancies statistical significances (P < 0.05) among the groups except Groups 1 and 2. |
| Shely et al. 2023 [26] | Mean ± SD, mm: ZZ: #15: 0.057±0.007 #16: 0.135±0.007 #17: 0.021±0.007 MIS: #15: 0.020±0.009 #16: 0.050±0.013 #17: 0.074±0.018 | Mean ± SD, degrees: ZZ: #15: 0.294±0.084 #16: 1.457±0.077 #17:0.139±0.059 MIS: #15: 0.400±0.251 #16: 1.776±0.464 #17:2.042±0.451 | R difference: Significant differences were found between MIS and ZZ.  #15 (p = 0.0001),  #16 (p = 0.0001),  #17(p = 0.0001). The mean error for MIS was lower in #15 and #16 than that for ZZ, in contrast to #17, which was higher for MIS. Ɵ difference: Significant differences were found between MIS and ZZ.  #15: p = 0.05,  #16: p = 0.001, #17: p = 0.0005. |
| Eldabe et al. 2025 [27] | 3D deviations ( Mean ± SD) (μm): TMSB: 61.46 ± 42.12  CSB:97.97 ± 56.69 | Angular deviations (Mean ± SD) (°): TMSB: 0.85 ± 0.69  CSB: 1.3 ± 1.06 | R difference: TMSB revealed a significantly lower linear deviation than conventional scan body (p = 0.005). Ɵ difference: TMSB revealed a significantly lower angular deviation than conventional scan body(p = 0.033). |
| Anwar et al. 2024 [28] | Mean ±SD, mm: NM: 0.282 ± 0.038 M: 0.229 ±0.047 | NA | The modified scan bodies showed a statistically significant effect on the trueness (P=.004). |

Abbreviations: SD: Standard deviation; IQR: Interquartile range; NA: Not available; RMS: Root mean square; µm: Micrometer; mm: Millimeter; °: Degrees; Ø: Diameter; ICCI: Internal conical connection implant; R difference: Linear displacement difference; Ɵ difference: Angular displacement difference.

**Table S3.** Results of the quality assessment of the included studies.

| **Number** | **Study ID** | **Clearly stated aims/objectives** | **Detailed explanation of sample size calculation** | **Detailed explanation of sampling technique** | **Detail of comparison group** | **Detailed explanation of methodology** | **Operator details** | **Randomization** | **Method of measurement of outcome** | **Outcome assessor details** | **Blinding** | **Statistical analysis** | **Presentation of results** | **Total Scores** | **%RoB** | **Interpretation** |
| --- | --- | --- | --- | --- | --- | --- | --- | --- | --- | --- | --- | --- | --- | --- | --- | --- |
| 1 | Mizumoto et al. 2020 [12] | 2 | 0 | NA | 2 | 2 | 0 | 1 | 2 | 0 | 0 | 2 | 2 | 13 | 59.09091 | Medium risk |
| 2 | Jung et al. 2022 [13] | 2 | 0 | NA | 2 | 2 | 0 | 0 | 2 | 0 | 0 | 2 | 2 | 12 | 54.54545 | Medium risk |
| 3 | Moslemion et al. 2020 [14] | 2 | 2 | NA | 2 | 2 | 2 | 0 | 2 | 0 | 0 | 2 | 2 | 16 | 72.72727 | Low risk |
| 4 | Pan et al. 2022 [1] | 2 | 2 | NA | 2 | 2 | 0 | 0 | 2 | 0 | 0 | 2 | 2 | 14 | 63.63636 | Medium risk |
| 5 | Lawand et al. 2024 [10] | 2 | 0 | NA | 2 | 2 | 0 | 0 | 2 | 0 | 0 | 2 | 2 | 12 | 54.54545 | Medium risk |
| 6 | Alvarez et al. 2022 [11] | 2 | 0 | NA | 2 | 2 | 2 | 0 | 2 | 0 | 0 | 2 | 2 | 14 | 63.63636 | Medium risk |
| 7 | Motel et al. 2020 [2] | 2 | 0 | NA | 2 | 2 | 0 | 0 | 2 | 0 | 0 | 2 | 2 | 12 | 54.54545 | Medium risk |
| 8 | Huang et al. 2021 [3] | 2 | 0 | NA | 2 | 2 | 2 | 0 | 2 | 0 | 0 | 2 | 2 | 14 | 63.63636 | Medium risk |
| 9 | Huang et al. 2020 [4] | 2 | 0 | NA | 2 | 2 | 2 | 0 | 2 | 0 | 0 | 2 | 2 | 14 | 63.63636 | Medium risk |
| 10 | Revilla-León et al. 2020 [5] | 2 | 0 | NA | 2 | 2 | 0 | 0 | 2 | 0 | 0 | 2 | 2 | 12 | 54.54545 | Medium risk |
| 11 | Revilla-León et al. 2021 [6] | 2 | 0 | NA | 2 | 2 | 0 | 0 | 2 | 0 | 0 | 2 | 2 | 12 | 54.54545 | Medium risk |
| 12 | Meneghetti et al. 2023 [7] | 2 | 1 | NA | 2 | 2 | 2 | 0 | 2 | 0 | 0 | 2 | 2 | 15 | 68.18182 | Medium risk |
| 13 | Ramadan et al. 2023 [8] | 2 | 2 | NA | 2 | 2 | 0 | 0 | 2 | 0 | 0 | 2 | 2 | 14 | 63.63636 | Medium risk |
| 14 | Yilmaz et al. 2021 [9] | 2 | 1 | NA | 2 | 2 | 2 | 0 | 2 | 0 | 0 | 2 | 2 | 15 | 68.18182 | Medium risk |
| 15 | Schmidt et al. 2021 [15] | 2 | 0 | NA | 2 | 2 | 0 | 0 | 2 | 0 | 0 | 2 | 2 | 12 | 54.54545 | Medium risk |
| 16 | Tan et al. 2022 [16] | 2 | 0 | NA | 2 | 2 | 0 | 1 | 2 | 0 | 0 | 2 | 2 | 13 | 59.09091 | Medium risk |
| 17 | Zhang et al. 2024 [18] | 2 | 0 | NA | 2 | 2 | 2 | 0 | 2 | 1 | 0 | 2 | 2 | 15 | 68.18182 | Medium risk |
| 18 | Anwar et al. 2024 [28] | 2 | 0 | NA | 2 | 2 | 2 | 0 | 2 | 0 | 0 | 2 | 2 | 14 | 63.63636 | Medium risk |
| 19 | Shely et al. 2023 [26] | 2 | 0 | NA | 2 | 2 | 0 | 0 | 2 | 0 | 0 | 2 | 2 | 12 | 54.54545 | Medium risk |
| 20 | Eldabe et al.2025 [27] | 2 | 0 | NA | 2 | 2 | 0 | 0 | 2 | 0 | 0 | 2 | 2 | 12 | 54.54545 | Medium risk |
| 21 | Alkindi et al. 2024 [19] | 2 | 0 | NA | 2 | 2 | 2 | 0 | 2 | 0 | 0 | 2 | 2 | 14 | 63.63636 | Medium risk |
| 22 | Park et al. 2024 [20] | 2 | 0 | NA | 2 | 2 | 0 | 0 | 2 | 0 | 0 | 2 | 2 | 12 | 54.54545 | Medium risk |
| 23 | Pan et al. 2025 [21] | 2 | 0 | NA | 2 | 2 | 0 | 0 | 2 | 0 | 0 | 2 | 2 | 12 | 54.54545 | Medium risk |
| 24 | Ashry et al. 2024 [22] | 2 | 0 | NA | 2 | 2 | 0 | 0 | 2 | 0 | 0 | 2 | 2 | 12 | 54.54545 | Medium risk |
| 25 | Li Y et al. 2024 [17] | 2 | 0 | NA | 2 | 2 | 2 | 0 | 2 | 0 | 0 | 2 | 2 | 14 | 63.63636 | Medium risk |
| 26 | Farah et al. 2025 [23] | 2 | 0 | NA | 2 | 2 | 1 | 0 | 2 | 0 | 0 | 2 | 2 | 13 | 59.09091 | Medium risk |
| 27 | Michelinakis et al. 2024 [24] | 2 | 0 | NA | 2 | 2 | 1 | 0 | 2 | 0 | 0 | 2 | 2 | 13 | 59.09091 | Medium risk |
| 28 | Uzel et al. 2023 [25] | 2 | 0 | NA | 2 | 2 | 0 | 0 | 2 | 0 | 0 | 2 | 2 | 12 | 54.54545 | Medium risk |

**Reference**

1. Pan, Y.; Tsoi, J.K.H.; Lam, W.Y.H.; Chen, Z.; Pow, E.H.N. Does the geometry of scan bodies affect the alignment accuracy of computer-aided design in implant digital workflow: An in vitro study? *Clin Oral Implants Res* **2022**, *33*, 313–321, doi:10.1111/clr.13890.

2. Motel, C.; Kirchner, E.; Adler, W.; Wichmann, M.; Matta, R.E. Impact of Different Scan Bodies and Scan Strategies on the Accuracy of Digital Implant Impressions Assessed with an Intraoral Scanner: An In Vitro Study. *J Prosthodont* **2020**, *29*, 309–314, doi:10.1111/jopr.13131.

3. Huang, R.; Liu, Y.; Huang, B.; Zhou, F.; Chen, Z.; Li, Z. Improved accuracy of digital implant impressions with newly designed scan bodies: an in vivo evaluation in beagle dogs. *BMC Oral Health* **2021**, *21*, 623, doi:10.1186/s12903-021-01986-2.

4. Huang, R.; Liu, Y.; Huang, B.; Zhang, C.; Chen, Z.; Li, Z. Improved scanning accuracy with newly designed scan bodies: An in vitro study comparing digital versus conventional impression techniques for complete-arch implant rehabilitation. *Clin Oral Implants Res* **2020**, *31*, 625–633, doi:10.1111/clr.13598.

5. Revilla-León, M.; Fogarty, R.; Barrington, J.J.; Zandinejad, A.; Özcan, M. Influence of scan body design and digital implant analogs on implant replica position in additively manufactured casts. *J Prosthet Dent* **2020**, *124*, 202–210, doi:10.1016/j.prosdent.2019.07.011.

6. Revilla-León, M.; Smith, Z.; Methani, M.M.; Zandinejad, A.; Özcan, M. Influence of scan body design on accuracy of the implant position as transferred to a virtual definitive implant cast. *J Prosthet Dent* **2021**, *125*, 918–923, doi:10.1016/j.prosdent.2020.03.019.

7. Meneghetti, P.C.; Li, J.; Borella, P.S.; Mendonça, G.; Burnett, L.H., Jr. Influence of scanbody design and intraoral scanner on the trueness of complete arch implant digital impressions: An in vitro study. *PLoS One* **2023**, *18*, e0295790, doi:10.1371/journal.pone.0295790.

8. Ramadan, R.E.; Razek, M.K.A.; Mohamed, F.S.; Fahmy, R.A.; Abd-Ellah, M.E. Positional transfer accuracy of titanium base implant abutment provided by two different scan body designs: an invitro study. *BMC Oral Health* **2023**, *23*, 746, doi:10.1186/s12903-023-03399-9.

9. Yilmaz, B.; Gouveia, D.; Marques, V.R.; Diker, E.; Schimmel, M.; Abou-Ayash, S. The accuracy of single implant scans with a healing abutment-scanpeg system compared with the scans of a scanbody and conventional impressions: An in vitro study. *J Dent* **2021**, *110*, 103684, doi:10.1016/j.jdent.2021.103684.

10. Lawand, G.; Ismail, Y.; Revilla-León, M.; Tohme, H. Effect of implant scan body geometric modifications on the trueness and scanning time of complete arch intraoral implant digital scans: An in vitro study. *J Prosthet Dent* **2024**, *131*, 1189–1197, doi:10.1016/j.prosdent.2022.06.004.

11. Alvarez, C.; Domínguez, P.; Jiménez-Castellanos, E.; Arroyo, G.; Orozco, A. How the geometry of the scan body affects the accuracy of digital impressions in implant supported prosthesis. In vitro study. *J Clin Exp Dent* **2022**, *14*, e1008–e1014, doi:10.4317/jced.59948.

12. Mizumoto, R.M.; Yilmaz, B.; McGlumphy, E.A., Jr.; Seidt, J.; Johnston, W.M. Accuracy of different digital scanning techniques and scan bodies for complete-arch implant-supported prostheses. *J Prosthet Dent* **2020**, *123*, 96–104, doi:10.1016/j.prosdent.2019.01.003.

13. Jung, H.T.; Kim, H.Y.; Song, S.Y.; Park, J.H.; Lee, J.Y. Accuracy of implant impression techniques with a scannable healing abutment. *J Prosthet Dent* **2022**, *128*, 729–734, doi:10.1016/j.prosdent.2020.06.042.

14. Moslemion, M.; Payaminia, L.; Jalali, H.; Alikhasi, M. Do Type and Shape of Scan Bodies Affect Accuracy and Time of Digital Implant Impressions? *Eur J Prosthodont Restor Dent* **2020**, *28*, 18–27, doi:10.1922/EJPRD_1962Moslemion10.

15. Schmidt, A.; Dent, D.M.; Billig, J.W.; Schlenz, M.A.; Wöstmann, B.; Dent, M. The Influence of Using Different Types of Scan Bodies on the Transfer Accuracy of Implant Position: An In Vitro Study. *International Journal of Prosthodontics* **2021**, *34*, 254–260, doi:10.11607/ijp.6796.

16. Tan, J.Z.H.; Tan, M.Y.; See Toh, Y.L.; Wong, K.Y.; Tan, K.B.C. Three-dimensional positional accuracy of intraoral and laboratory implant scan bodies. *J Prosthet Dent* **2022**, *128*, 735–744, doi:10.1016/j.prosdent.2020.09.057.

17. Li, Y.; Fang, H.; Yan, Y.; Geng, W. Accuracy of intraoral scanning using modified scan bodies for complete arch implant-supported fixed prostheses. *J Prosthet Dent* **2024**, *132*, 994.e991–994.e998, doi:10.1016/j.prosdent.2024.07.015.

18. Zhang, T.; Yang, B.; Ge, R.; Zhang, C.; Zhang, H.; Wang, Y. Effect of a Novel 'Scan Body' on the In Vitro Scanning Accuracy of Full-Arch Implant Impressions. *Int Dent J* **2024**, *74*, 847–854, doi:10.1016/j.identj.2024.01.015.

19. Alkindi, S.; Hamdoon, Z.; Aziz, A.M. Effect of different impression coping and scan body designs on the accuracy of conventional versus digital implant impressions: An in vitro study. *J Dent* **2024**, *146*, 105045, doi:10.1016/j.jdent.2024.105045.

20. Park, G.S.; Chang, J.; Pyo, S.W.; Kim, S. Effect of scan body designs and internal conical angles on the 3-dimensional accuracy of implant digital scans. *J Prosthet Dent* **2024**, *132*, 190.e191–190.e197, doi:10.1016/j.prosdent.2024.04.008.

21. Pan, Y.; Dai, X.; Tsoi, J.K.; Lam, W.Y.; Pow, E.H. Effect of shape and size of implant scan body on scanning accuracy: An in vitro study. *J Dent* **2025**, *152*, 105498, doi:10.1016/j.jdent.2024.105498.

22. Ashry, A.; Abdelhamid, A.M.; Ezzelarab, S.; Khamis, M.M. Effect of using scan body accessories and inter-implant distances on the accuracy of complete arch implant digital impressions: An in vitro study. *Journal of Prosthodontics* **2024**, doi:10.1111/jopr.13856.

23. Farah, R.I.; Alresheedi, B.; Alazmi, S.; Al-Haj Ali, S.N. Evaluating the impact of scan body angulation and geometric attachments on the accuracy of complete-arch digital implant impressions: A comparison of two intraoral scanners. *J Prosthodont* **2025**, *34*, 174–181, doi:10.1111/jopr.13807.

24. Michelinakis, G.; Apostolakis, D.; Nikolidakis, D.; Lapsanis, G. Influence of different scan body design features and intraoral scanners on the congruence between scan body meshes and library files: An in vitro study. *J Prosthet Dent* **2024**, *132*, 454.e451–454.e411, doi:10.1016/j.prosdent.2024.05.016.

25. Uzel, S.M.; Guncu, M.B.; Aktas, G.; Arikan, H.; Reiss, N.; Turkyilmaz, I. Influence of the implant scan body modifications on trueness of digital impressions. *JOURNAL OF DENTAL SCIENCES* **2023**, *18*, 1771–1777, doi:10.1016/j.jds.2023.04.004.

26. Shely, A.; Lugassy, D.; Rosner, O.; Zanziper, E.; Nissan, J.; Rachmiel, S.; Khoury, Y.; Ben-Izhack, G. The Influence of Laboratory Scanner versus Intra-Oral Scanner on Determining Axes and Distances between Three Implants in a Straight Line by Using Two Different Intraoral Scan Bodies: A Pilot In Vitro Study. *Journal of Clinical Medicine* **2023**, *12*, doi:10.3390/jcm12206644.

27. Eldabe, A.K.; Adel-Khattab, D.; Botros, K.H. Trueness of tooth modified scan bodies as a novel technique for edentulous full arch implant supported dental prosthesis: an in vivo prospective comparative study. *BMC Oral Health* **2025**, *25*, 29, doi:10.1186/s12903-024-05172-y.

28. Anwar, H.; Azer, A.; AboElHassan, R.G. Influence of a specially designed geometric device and modified scan bodies on the accuracy of a maxillary complete arch digital implant scan: An in vitro study. *J Prosthet Dent* **2024**, *131*, 683.e681–683.e687, doi:10.1016/j.prosdent.2024.02.020.
